# Efficient apoptosis and necrosis induction by proteasome inhibitor: bortezomib in the DLD-1 human colon cancer cell line

**DOI:** 10.1007/s11010-014-2216-y

**Published:** 2014-10-08

**Authors:** Rafał Krętowski, Anna Stypułkowska, Marzanna Cechowska-Pasko

**Affiliations:** Department of Pharmaceutical Biochemistry, Medical University of Bialystok, Mickiewicza 2A, 15-222 Bialystok, Poland

**Keywords:** Apoptosis, Bortezomib, DLD-1, Necrosis, ORP150, NF-κB

## Abstract

The inhibition of the 26S proteasome evokes endoplasmic reticulum stress, which has been shown to be implicated in the antitumoral effects of proteasome inhibitors. The cellular and molecular effects of the proteasome inhibitor—bortezomib—on human colon cancer cells are as yet poorly characterized. Bortezomib selectively induces apoptosis in some cancer cells. However, the nature of its selectivity remains unknown. Previously, we demonstrated that, in contrast to normal fibroblasts, bortezomib treatment evoked strong effect on apoptosis of breast cancer cells incubated in hypoxic and normoxic conditions. The study presented here provides novel information on the cellular effects of bortezomib in DLD-1 colon cancer cells line. We observe twofold higher percentage of apoptotic cells incubated for 48 h with 25 and 50 nmol/l of bortezomib in hypoxic conditions and four-, fivefold increase in normoxic conditions in comparison to control cells, incubated without bortezomib. It is of interest that bortezomib evokes strong effect on necrosis of DLD-1 colon cancer cell line. We observe the sixfold increase in necrosis of DLD-1 cells incubated with 25 or 50 nmol/l of bortezomib for 48 h in hypoxia and fourfold increase in normoxic conditions in comparison to adequate controls. We suggest that bortezomib may be candidates for further evaluation as chemotherapeutic agents for human colon cancer.

## Introduction

The proteasome is responsible for the degradation of short-lived proteins involved in gene expression, cell cycle progression, DNA repair, apoptosis, and signal transduction, as well as abnormal and misfolded proteins [1]. Proteasome-mediated protein degradation involves the covalent attachment of ubiquitin to specific proteins forming polyubiquitin chains, followed by the recruitment of these proteins to the proteasome for degradation [2]. The most common form of the 26S proteasome consists of one 20S core particle structure (700 kDa), which contains the protease activity, and two 19S regulatory caps (900 kDa each) [3]. There are three types of proteolytic activity within the 20S core particle: caspase-like, trypsin-like, and chymotrypsin-like activities [4].

The inhibition of the proteasome results in many toxic effects, including increased levels of ROS and the accumulation of unfolded and damaged proteins [5–7]. In response to proteasome inhibition, the cell induces specific protective mechanisms, including the unfolded protein response [5], autophagy [8, 9], and, if the damage is severe, apoptosis [5, 10, 11].

Bortezomib (PS-341; Velcade™), a highly selective and reversible inhibitor of the 26S proteasome approved for clinical use against multiple myeloma, is in clinical trials as a single agent or in combination with chemotherapeutics against other solid tumor malignancies [12]. The chemical name for bortezomib is [(1*R*)-3-methyl-1-({(2*S*)-3-phenyl-2-[(pyrazin-2-ylcarbonyl)amino]pro-panoyl}amino)-butyl] boronic acid. The mechanisms involved in its anti-cancer activity are still being elucidated, but evidence suggests that it inhibits proteasome degradation of IκB, an inhibitor of nuclear factor-κB (NF-κB) in the cancer cell [13–16]. Recently, bortezomib was shown to induce ER stress and ER-dependent apoptosis by blocking the ERAD system thereby promoting the accumulation of misfolded proteins in the ER that induce proteotoxicity and cell death [17, 18].

Rapidly proliferating cancer cells require increased ER activity to facilitate the folding, assembly, and transport of membrane and secretory proteins, and are thereby subjected to ER stress. Due to inadequate vascularization and rapid growth, tumor cells encounter growth-limiting conditions such as hypoxia and nutrient deprivation [19]. Tumor hypoxia is an important feature of the tumor microenvironment which is the factor promoting cancer progression, decreased response to therapy, and a poor patient survival [20]. Hypoxic tumor cells activate hypoxia inducible factor (HIF)-dependent and HIF-independent survival mechanisms that promote cancer progression [21, 22].

ER stress occurs when the protein load exceeds the ER capacity to fold or degrade them and is manifested by the accumulation of malfolded proteins in the endoplasmic reticulum. ER stress triggers a quality control mechanism, the unfolded protein response (UPR), which aims at restoring ER homeostasis by activating a cascade of signaling molecules to transiently arrest protein translation, to induce ER molecular chaperones and enzymes that enhance the protein folding capacity, and to initiate a process to export and degrade the misfolded ER proteins [23, 24].

One arm of the unfolded protein response is PERK-eIF2α-ATF4 pathway. In addition to PERK, UPR signaling is mediated by two other ER transmembrane proteins: IRE1 and ATF6. Upon ER stress, active IRE1 processes XBP-1 mRNA, removing a 26-nucleotide intron to generate a spliced XBP-1 mRNA encoding the functional XBP-1 transcription factor [25, 26]. Together, these pathways coordinately upregulate transcription of UPR target genes, such as the ER chaperone BiP/GRP78 and proteins involved in ER-associated degradation (ERAD), which aid in restoring ER homeostasis. The ERAD system shuttles misfolded proteins from the ER lumen to the cytosol where they become ubiquitinated and degraded by the 26S proteasome [27]. However, IRE1 and another UPR target, CHOP, are also involved in ER stress-induced apoptosis [28]. Thus the UPR, while activated as a pro-survival response under moderate or intermittent ER stress, can also lead to cell death under conditions of acute or chronic stress [24].

Apoptosis plays a major role in the control of cancer development. In fact, cells encounter multiple apoptotic stimuli during cancer progression, including hypoxia or nutrient deprivation. The function of this process is to eliminate unwanted or injured cells with characteristic cellular and biochemical hallmarks. Accordingly it has been suggested that the well-documented anti-apoptotic potential of chaperones may play a critical role in the suppression of apoptosis in cancer cells [29, 30].

While one attractive approach to target the UPR in tumors is to develop inhibitors against its effectors (against PERK or IRE1), we postulated that another approach could take advantage of its pro-death activity under extreme or prolonged stress. We decided to study the effect of bortezomib and ER stress, evoked by hypoxia, on apoptosis and necrosis of colon cancer cell line—DLD-1.

## Materials and methods

### Reagents

Dulbecco’s modified Eagle’s medium (DMEM), containing glucose at 4.5 mg/ml (25 mM) with Glutamax, penicillin, streptomycin, trypsin–EDTA were provided by Invitrogen (San Diego, USA), passive lysis buffer by Promega (Madison, USA), FBS Gold by Gibco (San Diego, USA), bortezomib by Selleckchem (Houston, USA), BCA Protein Assay Kit by Thermo Scientific (Rockford, USA), PE Annexin V Apoptosis Detection Kit I by BD Pharmingen^TM^ (CA, USA), Senescence Detection Kit by bioVision (CA, USA), Sigma-Fast BCIP/NBT reagent by Sigma (St Louis, MO, USA), Precision Plus Protein Standards dual color by Bio-Rad (Hercules, USA), monoclonal (mouse) anti-human ORP150 antibody by IBL (Gunma, Japan), monoclonal (mouse) anti-HIF-1α antibody by BD Biosciences (CA, USA), polyclonal (rabbit) NF-κB2 p100/p52 antibody by Cell Signaling Technology (Boston, USA), alkaline phosphatase-labeled anti-mouse immunoglobulin G by Rockland (PA, USA), and anti-rabbit IgG HRP-linked antibody by Cell Signaling Technology (Boston, USA).

### Cell cultures

Human colon cancer cell line DLD-1 was obtained from American Type Culture Collection (ATCC). The cells were maintained in high-glucose DMEM supplemented with 10 % heat-inactivated fetal bovine serum GOLD (FBS GOLD), 2 mM l-glutamine, penicillin (100 U/ml), and streptomycin (100 μg/ml). DLD-1 cells were cultured in Falcon flasks (BD) in a 5 % CO_2_ incubator (Galaxy S+; New Brunswick) at 37 °C. Subconfluent cultures were detached with 0.05 % trypsin, 0.02 % EDTA in calcium-free phosphate-buffered saline (PBS), and counted in a Scepter cell counter (Millipore), and then 5 × 10^5^ cells were seeded in six-well plates in 2 ml of growth medium for determination of protein concentration. In these conditions, they reached 70–80 % confluence. After incubation, the culture media were removed; the cells were washed with PBS and submitted to the action of lysis buffer for determination of protein concentration. It allowed the detachment of the cells and extracellular matrix from the bottom of culture vessels and their suspension in the buffer. The cells incubated on Petri dishes were exposed to hypoxic conditions.

### Cell viability

Cell viability was measured according to the method of Carmichael [31] using 3-(4,5-dimethylthiazol-2-yl)-2,5-diphenyltetrazolium bromide (MTT). Briefly, cells were seeded in 24-well plate at a density of 100,000 per well. Confluent cells, cultured for 12, 24, and 48 h in normoxic conditions with different concentrations of bortezomib, were washed three times with PBS and then incubated with 1 ml of MTT solution (0.25 mg/ml in PBS) for 4 h at 37^o^ C in 5 % CO_2_ in an incubator. The medium was removed and 1 ml of 0.1 mol/l HCl in absolute isopropanol was added. Absorbance of converted dye in living cells was measured at the wavelength of 570 nm. The viability of DLD-1 cells cultured in hypoxic conditions was calculated as the percentage of control cells incubated in normoxia. All the experiments were done in duplicate in at least three cultures.

### Induction of hypoxia in cell cultures

The cells (5 × 10^5^ in 2 ml of medium) were seeded in six-well plates and incubated until they achieved confluence. The high-glucose DMEM was removed and replaced with 2 ml of the same fresh medium with 25 or 50 nmol/l of bortezomib. Control cell cultures were kept in normoxic conditions whereas the tested cells were incubated in hypoxic conditions. Hypoxia was evoked by 12, 24, and 48 h incubation of cells in atmosphere containing a reduced to 1 % oxygen in hypoxia chamber (Galaxy 170R; New Brunswick an Eppendorf company). After incubation, the culture media were removed; the cell layers were washed with PBS and submitted to the action of lysis buffer for the determination of ORP150/GRP170, HIF-1α, NF-κB2 p100/p52 expression and protein assay. It allowed the separation of cells and extracellular matrix from the bottom of culture vessels and their suspension in the buffer. The cells incubated on six-well plates were detached with trypsin and analyzed by flow cytometry method.

### Detection of apoptosis

Cells were incubated in the high-glucose DMEM in hypoxic and normoxic conditions with different concentrations of bortezomib. The incubation was continued for 12, 24, and 48 h. Apoptosis was evaluated by flow cytometry on FACSCanto II cytometer (Becton Dickinson). The cells were trypsinized, resuspended in DMEM, and then in binding buffer. Cells were stained with FITC Annexin V and PI for 15 min at room temperature in the dark following the manufacturer’s instructions (FITC Annexin V apoptosis detection Kit I). Data were analyzed with FACSDiva software and dead cells were excluded based on forward- and side-scatter parameters.

### Sodium dodecyl sulfate/Polyacrylamide gel electrophoresis (SDS/PAGE)

Cells were washed with cold PBS and solubilized in 100 μl of passive lysis buffer per well. The lysates were centrifuged for 10 min at 12,000×*g* at 4 °C. Samples of lysates containing 20 μg of protein were subjected to SDS–PAGE, as described by Laemmli [32]. The Bio-Rad Precision Plus Protein Standards dual color were used. The electrophoresis was run for 40–45 min. In each experiment, 7.5 % polyacrylamide gel and constant current (25 mA) were used.

### Immunoblotting

The proteins were transferred to nitrocellulose membranes and then pre-treated for 2 h with Tris-buffered saline (TBS) containing 0.05 % Tween 20 (TBS-T) and 5 % non-fat dry milk, at room temperature. Membranes were probed for 16 h with a mixture containing monoclonal (mouse) anti-human ORP150 antibody (1:100) or monoclonal (mouse) anti-human HIF-1α (1:500) or polyclonal (rabbit) NF-κB2 p100/p52 antibody (1:1,000) in 5 % dried milk in TBS-T, at 4 °C. Then the alkaline phosphatase-conjugated antibody against mouse IgG at 1:2,500 dilution or anti-rabbit IgG HRP-linked (1:1,000) was added for 1 h in TBS-T with slow shaking. The nitrocellulose was washed with TBS-T (five times for 5 min) and exposed to Sigma-Fast BCIP/NBT reagent.

### Protein assay

Protein concentration in cell lysates was determined by the method of Smith et al. [33] using BCA Protein Assay Kit (Thermo Scientific, USA). Bovine serum albumin was used as a standard.

### Statistical analysis

Mean values from three independent experiments ± standard deviations (SD) were calculated. Statistical analysis was performed using Student’s *t* test.

## Results

### The effect of bortezomib on viability of DLD-1 cell line

**Fig. 1:**
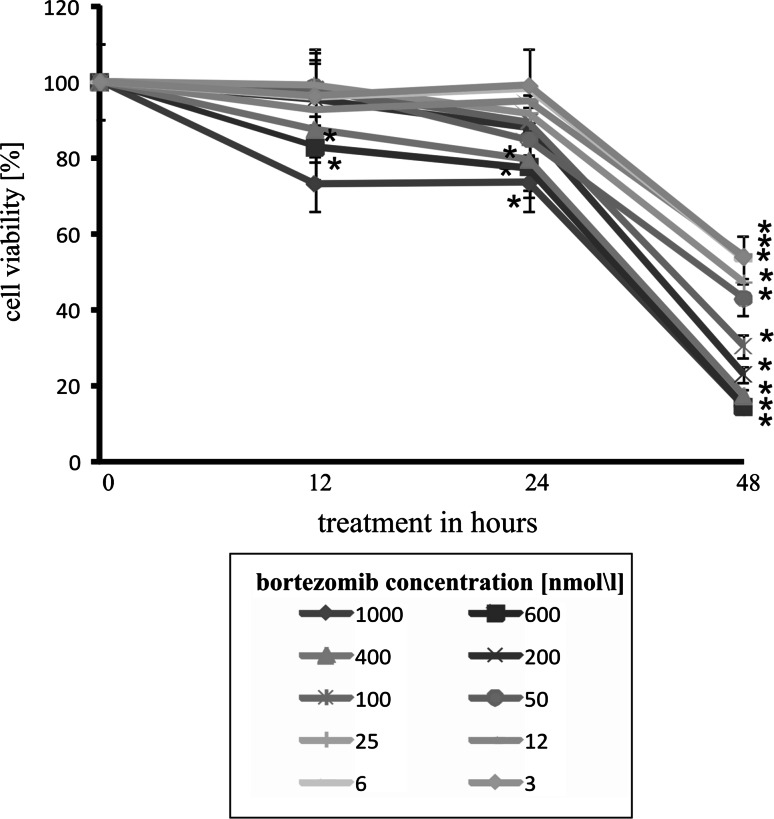
The viability of DLD-1 cells treated with different concentrations of bortezomib for 12, 24, and 48 h. Mean values from three independent experiments ± SD are presented. Significant alterations are expressed relative to controls and marked with asterisks. Statistical significance was considered if * *p* < 0.05

The antiproliferative effect of bortezomib was assessed by MTT method in DLD-1 cells cultured with increasing concentrations of bortezomib for periods of 12, 24, or 48 h. Figure 1 shows that bortezomib, in the concentration from 3 to 1,000 nmol/l, caused a time-dependent and dose-dependent strong reduction in cell viability of the colon cancer DLD-1 cells. An evident inhibition in cell viability was observed as early as after 24 h. In cells treated with higher concentrations of bortezomib, the effect on cell viability was markedly more pronounced (Fig. 1). These results show that bortezomib exhibits a time-dependent and dose-dependent evident inhibition in cell viability of colon cancer DLD-1 cells. Two concentrations of bortezomib (25 and 50 nmol/l) were chosen for further study. Both were up to the value of the half maximal inhibitory concentration (IC_50_) for bortezomib.

### Detection of HIF-1α in DLD-1 cells submitted to hypoxia

**Fig. 2:**
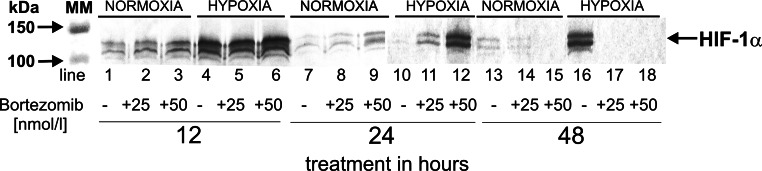
Western blot analysis of HIF-1α synthesized by DLD-1 cells incubated in normoxic and hypoxic conditions for 12, 24 and 48 h. Samples containing 20 μg of protein were submitted to electrophoresis and immunoblotting. A representative Western blot is presented. The molecular mass (MM) of the precision plus protein standards are indicated on the left side of the lanes

We also characterized the expression of HIF-1α, a biochemical marker of hypoxia. Figure 2 shows that DLD-1cells grown in normoxic conditions with or without bortezomib (for 12 h—lanes 1–3; for 24 h—lanes 7–9; for 48 h—lanes 13–15) demonstrate a weak expression of HIF-1α. In contrast, those cells incubated in hypoxic conditions demonstrated an intense expression of HIF-1α after 12 h of incubation with or without bortezomib (Fig. 2, lanes 4–6). After 24 h without bortezomib, we observe a weak expression of HIF-1α (Fig. 2, lane 10) and strong expression with bortezomib, especially with 50 nmol/l concentration (Fig. 2, lane 12). In contrast to 12 and 24 h, after 48 h incubation of DLD-1 cells in hypoxic conditions without bortezomib, we observe an intense expression of HIF-1α (Fig. 2, lane 16). It is worthy of note that incubation of these cells in hypoxic conditions with bortezomib did not demonstrate the HIF-1α expression with 25 and 50 nmol/l of bortezomib (Fig. 2, lanes 17, 18). Additionally, the expression of HIF-1α after 12 h in hypoxic conditions with bortezomib (lanes 5, 6) was higher in comparison to the same cells incubated for 24 (lanes 11, 12) or 48 h (lanes 17, 18).

### The effect of bortezomib on apoptosis of DLD-1 cells submitted to hypoxia

**Fig. 3:**
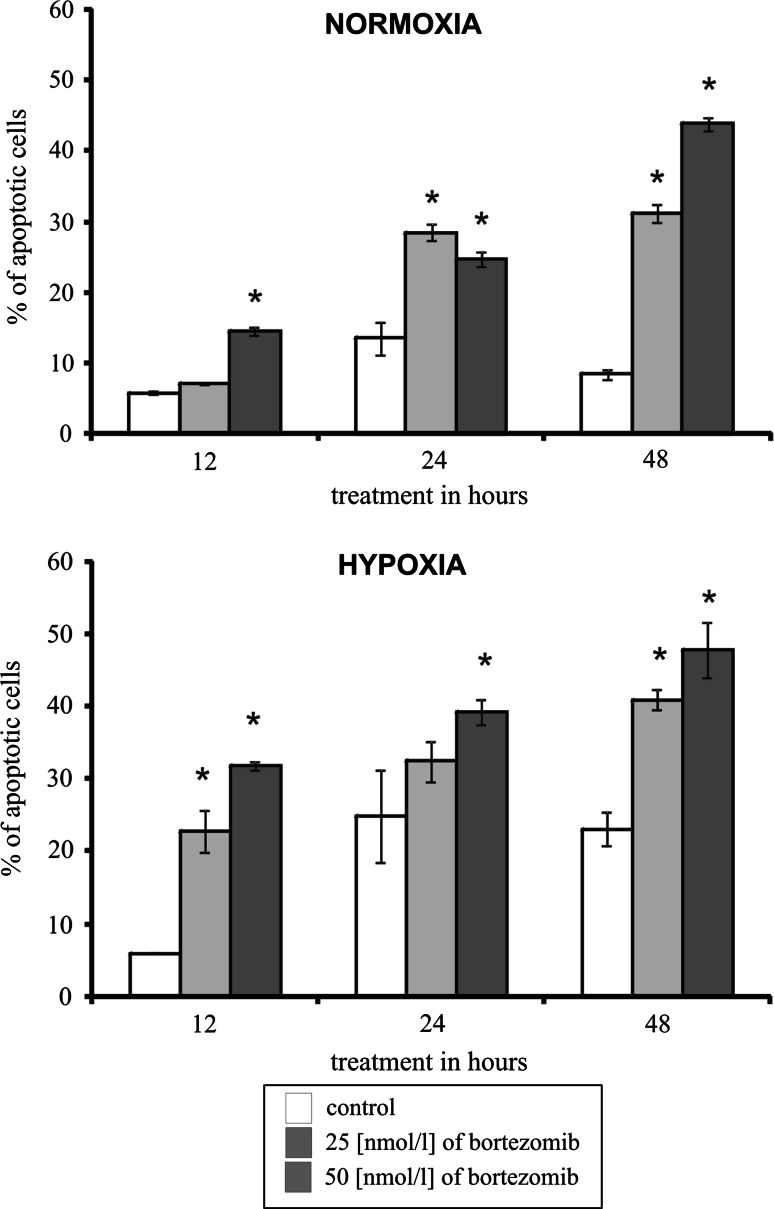
The effect of bortezomib on apoptosis of DLD-1cells incubated in normoxic and hypoxic conditions for 12, 24 and 48 h. Mean values from three independent experiments ± SD are presented. Significant alterations are expressed relative to controls and marked with asterisks. Statistical significance was considered if *p < 0.05

Next, we investigated whether bortezomib toxicity was due to the induction of apoptosis. Figure 3 shows the per cent of apoptotic DLD-1 cells incubated for 12, 24, or 48 h in normoxic and hypoxic conditions with 25 or 50 nmol/l of bortezomib. In the cells incubated for 12, 24, and 48 h in hypoxic and normoxic conditions with bortezomib, we observed a time-dependent and dose-dependent increase in apoptosis of DLD-1 cells (Fig. 3). The per cent of apoptotic cells after 12, 24, and 48 h of incubation was significantly higher for 50 nmol/l of bortezomib in comparison to control cells. We observe twofold higher percentage of apoptotic cells incubated for 48 h with 25 and 50 nmol/l of bortezomib in hypoxic conditions and 3, 4-fold higher in normoxic conditions in comparison to adequate control cells, incubated without bortezomib. The per cent of apoptotic cells in the colon cancer cells cultured in hypoxia was higher in comparison to the same cells incubated in normoxia, notwithstanding the incubation time and concentration of bortezomib (Fig. 3).

### The effect of bortezomib on necrosis of DLD-1 cells submitted to hypoxia

**Fig. 4:**
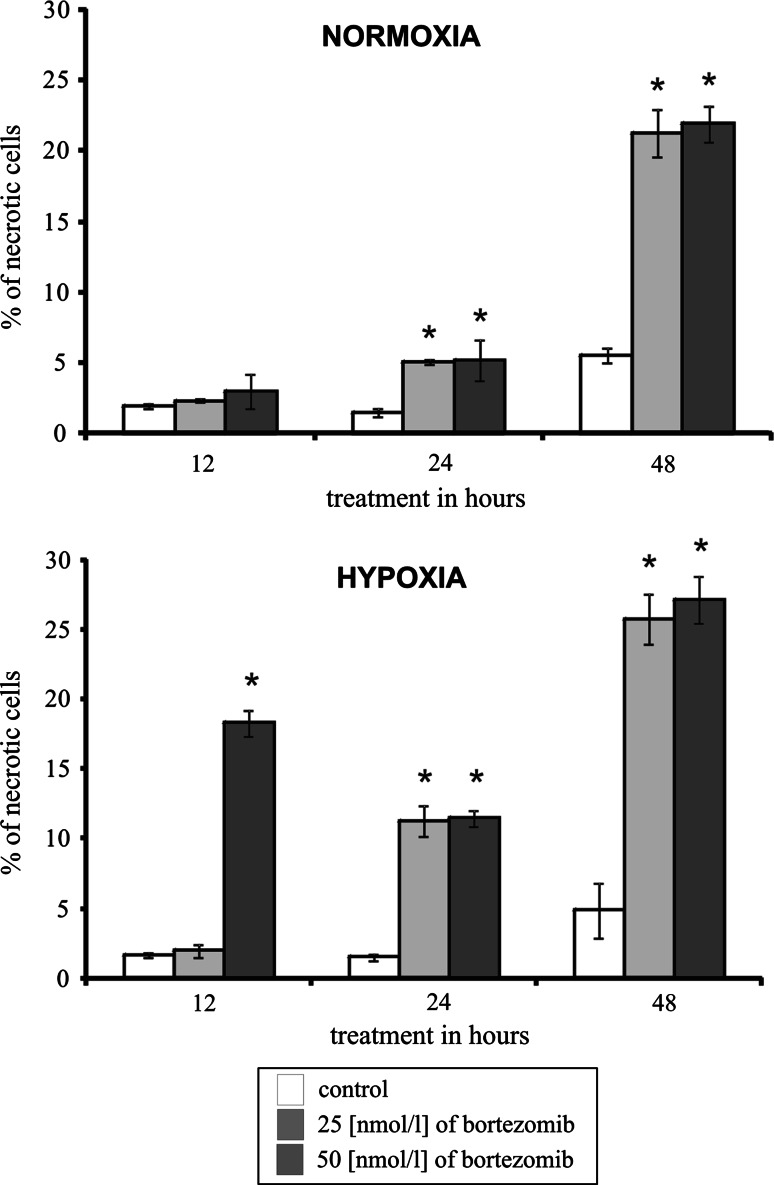
The effect of bortezomib on necrosis of DLD-1 cells incubated in normoxic and hypoxic conditions for 12, 24 and 48 h. Mean values from three independent experiments ± SD are presented. Significant alterations are expressed relative to controls and marked with asterisks. Statistical significance was considered if * *p* < 0.05

Next, we investigated whether bortezomib toxicity was due to the induction of necrosis. Figure 4 shows the per cent of necrotic DLD-1 cells incubated for 12, 24 or 48 h in normoxic and hypoxic conditions with 25 or 50 nmol/l of bortezomib. In the cells incubated for 12, 24, and 48 h in hypoxic and normoxic conditions with bortezomib, we observed a time-dependent increase in necrosis of DLD-1 cells (Fig. 4). The per cent of necrotic cells after 12 h for 50 nmol/l and after 24 and 48 h of incubation for both concentrations of bortezomib was significantly higher in comparison to adequate control cells. We observe the fourfold increase in necrosis of DLD-1 cells incubated with 25 or 50 nmol/l of bortezomib for 48 h in normoxia and sixfold increase in hypoxic conditions in comparison to adequate control. After 24 and 48 h, the per cent of necrotic cells in the colon cancer cells cultured in hypoxia was twofold higher in comparison to the same cells incubated in normoxia, notwithstanding the concentration of bortezomib (Fig. 4).

### The effect of bortezomib on the expression of ORP150

**Fig. 5:**
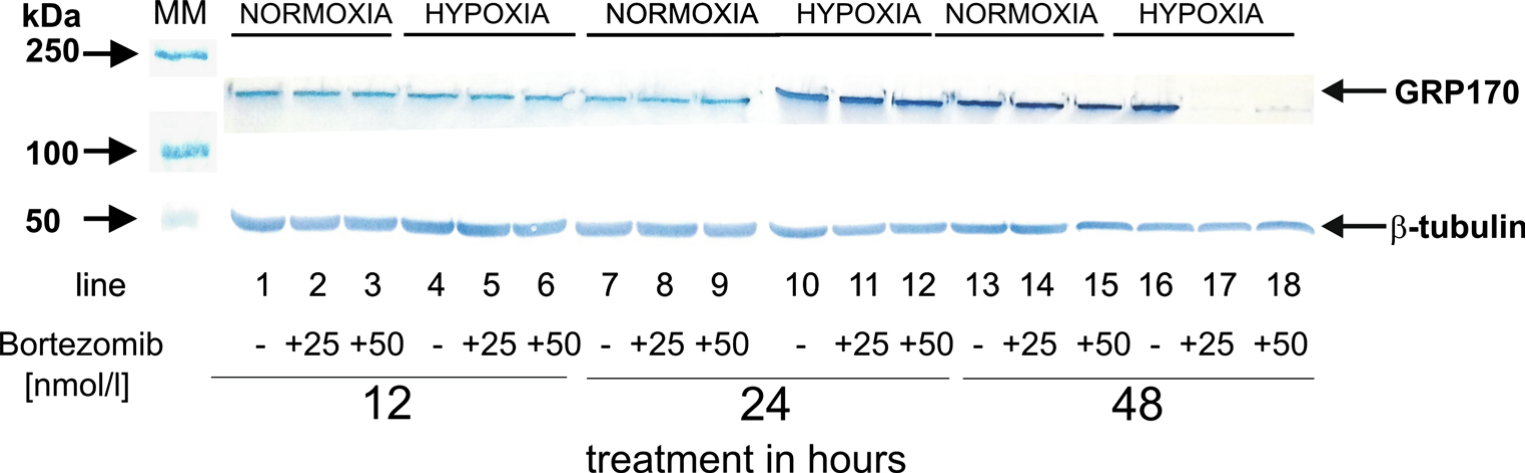
Western blot analysis of ORP150/GRP170 synthesized in DLD-1 cells incubated with 25 and 50 nmol/l of bortezomib in normoxic and hypoxic conditions for 12, 24 and 48 h. Samples containing 20 μg of protein were submitted to electrophoresis and immunoblotting. A representative Western blot is presented. The molecular mass (MM) of the precision plus protein standards are indicated on the left side of the lanes

Overexpression of Hsp70 chaperones (ORP150 belongs to this family) may protect cancer cells against entering the apoptotic pathway. Figure 5 shows the expression of GRP170, glycosylated form of ORP150, in DLD-1 incubated in normoxic and hypoxic conditions with 25 and 50 nmol/l of bortezomib.

 The expression of GRP170 in DLD-1 was observed in cultures incubated in normoxic and hypoxic conditions for 12 and 24 h, notwithstanding the concentration of bortezomib (Fig. 5; lanes 1–12). We did not observe the induction of ORP150 expression in hypoxic conditions in DLD-1 incubated for 12, 24 and 48 h, notwithstanding the concentration of bortezomib (Fig. 5; lanes: 1–18). It is of interest that DLD-1 cells incubated for 48 h in hypoxic conditions with 25 and 50 nmol/l of bortezomib did not express both GRP170 and ORP150 (Fig. 5; lanes 17 and 18), while without bortezomib, we observed a strong expression of GRP170 (Fig. 5; lane 16).

### The effect of bortezomib on the expression of NF-κB in DLD-1 cells submitted to hypoxia

**Fig. 6:**
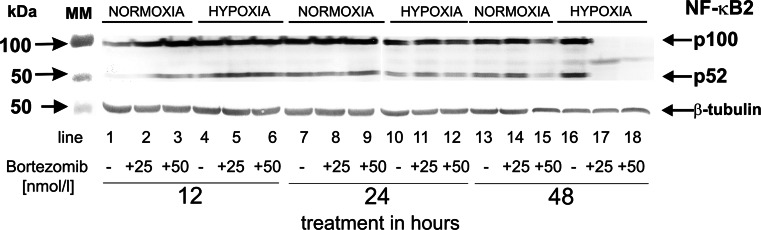
Western blot analysis of NF-κB synthesized in DLD-1 cells incubated with 25 and 50 nmol/l of bortezomib in normoxic and hypoxic conditions for 12, 24 and 48 h. Samples containing 20 μg of protein were submitted to electrophoresis and immunoblotting. A representative Western blot is presented. The molecular mass (MM) of the precision plus protein standards are indicated on the left side of the lanes

We also characterized the expression of NF-κB2, the factor involved in suppression of apoptosis. The expression of NF-κB in DLD-1 was observed in cultures incubated in normoxic conditions for 12, 24 and 48 h, notwithstanding the concentration of bortezomib (Fig. 6; lanes 1–3, lanes 7–9, lanes 13–15). The induction of NF-κB expression was observed in hypoxic conditions in DLD-1 incubated for 12 and 24 h, notwithstanding the concentration of bortezomib (Fig. 6; lanes: 4–6, 10–12). It is of interest that DLD-1 cells incubated for 48 h in hypoxic conditions with 25 or 50 nmol/l of bortezomib did not express NF-κB (Fig. 6; lanes 17 and 18).

## Discussion

We have investigated the effect of the proteasome inhibitor—bortezomib with the physiologic stress of hypoxia on tumor cells. The inhibition of the 26S proteasome evokes endoplasmic reticulum stress, which has been shown to be implicated in the antitumoral effects of proteasome inhibitors. The cellular and molecular effects of the proteasome inhibitor—bortezomib—on human colon cancer cells are as yet poorly characterized. Bortezomib selectively induces apoptosis in some cancer cells. However, the nature of its selectivity remains unknown. Previously, we demonstrated that in contrast to normal fibroblasts, bortezomib treatment evoked strong effect on apoptosis of breast cancer cells incubated in hypoxic and normoxic conditions [34]. The study presented here provides novel information on the cellular effects of bortezomib in colon cancer cells line DLD-1. We observe twofold higher percentage of apoptotic cells incubated for 48 h with 25 and 50 nmol/l of bortezomib in hypoxic conditions and 4-, 5-fold increase in normoxic conditions in comparison to control cells incubated without bortezomib.

Another form of cell death, necrosis, may be triggered by metabolic stress under conditions of defective apoptosis or prolonged autophagy. It is of interest that bortezomib evokes strong effect on necrosis of DLD-1 colon cancer cell line both in hypoxic and normoxic conditions. We observe the sixfold increase in necrosis of DLD-1 cells incubated with 25 or 50 nmol/l of bortezomib for 48 h in hypoxia and fourfold increase in normoxic conditions in comparison to adequate controls. Our findings suggest that it may be possible to “push” hypoxic tumor cells into necrosis by overactivating the ER stress-dependent mechanisms.

The precise mechanism of hypoxia-induced, ER-dependent cell death is still unknown. In agreement with other reports, one potential player may be the pro-apoptotic protein CHOP, induced by bortezomib, hypoxia and combined treatment [17, 18].

The maintenance of protein homeostasis in cell requires the activities of chaperones and the ubiquitin–proteasome system, which together serve to inactivate and degrade misfolded proteins. When proteins are not folded properly, they are directed to 26S proteasomal degradation. If misfolded or unfolded proteins are not degraded by the proteasome, they form aggregates and lead to the ER stress. The ER stress triggers UPR to reduce the accumulation of unfolded proteins and restore the ER function. When protein aggregation or ER stress persists, the UPR signaling switches from the pro-survival to pro-apoptotic. Consequently, the 26S proteasome complex also plays an important role in regulating the ER stress and cell survival. Therefore, inhibition of the proteasomal function in cancer cells would promote apoptosis and have an anti-tumor function [9]. In fact, the inhibition of the proteolytic activity of the 26S proteasome has been shown to induce pro-apoptotic ER stress in multiple myeloma [18], pancreatic [17], head and neck cancer [11], and non-small cell lung carcinoma [35].

Proteasomal activity is essential for eliminating excess proteins, and by counteracting protein production, it establishes steady protein levels [36]. The ubiquitin proteasome pathway represents the major pathway for intracellular protein degradation. The 26S proteasome is responsible for the degradation of approximately 80 % of cellular proteins, including misfolded and mutated proteins as well as those involved in the regulation of development, differentiation, cell proliferation, signal transduction, apoptosis, and antigen presentation [13].

Prolonged proteasome inhibition induces stress responses that initiate apoptosis via intrinsic pathway [36]. This is exploited clinically in the treatment of multiple myeloma with the proteasome inhibitor bortezomib. Inhibition of proteasome activity by bortezomib is associated with an accumulation and transcriptional induction of BH3-only proteins such as PUMA, BIM, NOXA, or BIK. BH3-only proteins antagonize anti-apoptotic BCL-2 family members such as BCL-2, BCL-xL, or Mcl-1 and can activate the pro-apoptotic members BAX and BAK [37]. Activated BAX and BAK form pores in the outer mitochondrial membrane, resulting in cytochrome *c* and Smac/Diablo release from the intermembrane space into the cytosol. This results in caspase-9 activation, inhibition of IAP (inhibitor of apoptosis proteins), and subsequent apoptosis execution by effector caspases [37]. Induction of NOXA has been reported to be a key mechanism in bortezomib-mediated apoptosis which is independent of P53 status but dependent on c-Myc [38–40]. Bortezomib-mediated apoptosis is accompanied by the induction of c-Jun-NH2 terminal kinase, generation of reactive oxygen species, release of cytochrome c, second mitochondria-derived activator of caspases, and apoptosis-inducing factor, and activation of the intrinsic caspase-9 pathway and extrinsic caspase-8 pathway [13].

In agreement with the cytoprotective role of molecular chaperones it has been shown, that they can prevent stress-induced apoptosis [29, 30]. Overexpression of Hsp70 chaperones (ORP150 belongs to this family) prevents cytochrome c release from mitochondria, blocks apoptosome formation by binding to the apoptotic protease-activating factor (Apaf-1), inhibits the release of apoptosis-inducing factor (AIF) from mitochondria, and prevents the loss of mitochondrial transmembrane potential. The AIF released from mitochondria binds to Hsp70 and this interaction makes impossible the nuclear import of AIF [30]. It is of interest that DLD-1 cells incubated for 48 h in hypoxic conditions with 25 and 50 nmol/l of bortezomib did not express both GRP170 and ORP150, while without bortezomib we observed strong expression of GRP170.

The transcription factor NF-κB is believed to play a vital role in the action of bortezomib as it is involved in the suppression of apoptosis and induction of cancer cell proliferation, invasion, metastasis, tumorigenesis, and angiogenesis [41]. NF-κB2 is heterodimer of p52 and p65. The 26S proteasome is involved in generating p52 from the precursor protein p100. Then p52 binds to p65 and becomes the active dimer of NF-κB2. In the cytosol, inhibitor of NF-κB (IκB) binds to NF-κB and inhibits the translocation of NF-κB to the nucleus for gene activation [41]. NF-κB is activated by proteasomal degradation of IκB. It is of interest that DLD-1 cells incubated for 48 h in hypoxic conditions with 25 and 50 nmol/l of bortezomib did not express NF-κB in contrast to the same cells incubated in normoxic conditions.

Proteasome inhibitor bortezomib promotes cell death in colon cancer cell line DLD-1 both through apoptosis and necrosis. We suggest that in the mechanism of DLD-1, cell death is involved in the suppression of chaperone GRP170 and inhibition of transcription factor NF-κB synthesis. Chaperone induction in cancer cells can lead to cancer progression and may be a major cause of chemotherapeutics resistance. It is one of the mechanisms protecting cancer cells against entering the apoptotic pathway. Hence, chaperone inhibition may be a promising tool to decrease cytoprotection and to initiate apoptosis of cancer cells [42].

In conclusion, bortezomib represents compounds, which are able to induce apoptosis and necrosis in human cancer cells in low dose. We suggest that bortezomib may be candidates for further evaluation as chemotherapeutic agents for human colon cancer.
